# Extracellular, cell-permeable survivin inhibits apoptosis while promoting proliferative and metastatic potential

**DOI:** 10.1038/sj.bjc.6604978

**Published:** 2009-03-17

**Authors:** S Khan, J R Aspe, M G Asumen, F Almaguel, O Odumosu, S Acevedo-Martinez, M De Leon, W H R Langridge, N R Wall

**Affiliations:** 1 Division of Biochemistry and Microbiology, Department of Basic Sciences, Center for Health Disparities Research and Molecular Medicine, Loma Linda University, Loma Linda, CA 92350, USA; 2 Division of Physiology and Pharmacology, Department of Basic Sciences, Center for Health Disparities Research and Molecular Medicine, Loma Linda University, Loma Linda, CA 92350, USA

**Keywords:** tumour microenvironment, survivin, apoptosis, metastasis

## Abstract

The tumour microenvironment is believed to be involved in development, growth, metastasis, and therapy resistance of many cancers. Here we show survivin, a member of the inhibitor of apoptosis protein (IAP) family, implicated in apoptosis inhibition and the regulation of mitosis in cancer cells, exists in a novel extracellular pool in tumour cells. Furthermore, we have constructed stable cell lines that provide the extracellular pool with either wild-type survivin (Surv-WT) or the previously described dominant-negative mutant survivin (Surv-T34A), which has proven pro-apoptotic effects in cancer cells but not in normal proliferating cells. Cancer cells grown in conditioned medium (CM) taken from Surv-WT cells absorbed survivin and experienced enhanced protection against genotoxic stresses. These cells also exhibited an increased replicative and metastatic potential, suggesting that survivin in the tumour microenvironment may be directly associated with malignant progression, further supporting survivin's function in tumourigenesis. Alternatively, cancer cells grown in CM taken from the Surv-T34A cells began to apoptose through a caspase-2- and caspase-9-dependent pathway that was further enhanced by the addition of other chemo- and radiotherapeutic modalities. Together our findings suggest a novel microenvironmental function for survivin in the control of cancer aggressiveness and spread, and should result in the genesis of additional cancer treatment modalities.

The complexity and heterogeneity of the tumour microenvironment (Tredan *et al*, 2007) is believed to have an important function in the development, growth, metastasis, and therapy resistance of many cancers (Ohtani, 1998). The tumour is not merely a single genotype-containing clonal mass but a community of heterogeneous cancer cells and stromal cells embedded in an extracellular matrix and nourished by vascular and fibroblast cell networks (Aznavoorian *et al*, 1990).

The multifunctional protein survivin controls diverse cellular functions, including surveillance checkpoints, suppression of cell death, the regulation of mitosis, and the adaptation to unfavourable environments (Li *et al*, 1998; Altieri, 2003, 2006). The suppression of cell death activities and baculovirus inhibitor of apoptosis protein (IAP) repeat (BIR) domain characterises survivin as a member of the IAP family (Ambrosini *et al*, 1997). However, survivin's lack of a C-terminal RING finger domain and a caspase recruitment domain (Deveraux and Reed, 1999) makes it structurally unique among the mammalian IAPs. The multifaceted functionality of survivin is still being intensely scrutinised, although it appears that protein compartmentalisation may be important. Survivin was recently shown to colocalise in the mitochondria, where it abolishes tumour cell apoptosis and promotes tumourigenesis in immunocompromised animals (Dohi *et al*, 2004). Survivin may therefore possess a function in apoptosis similar to the pro-apoptotic Bcl-2 family proteins. Furthermore, survivin has been also found in the nucleus and cytosol where the implication is that it has functions in mitosis regulation and apoptosis inhibition, respectively (Fortugno *et al*, 2002). Survivin is expressed in most common human cancers and although present during embryonic and foetal development, it is undetectable in a variety of adult tissues (Adida *et al*, 1998) and for this reason it is currently recognised as an important anticancer target (Fukuda and Pelus, 2006). For this purpose, multiple strategies have been successfully investigated, including the molecular antagonists such as antisense oligos, RNA inhibition, dominant-negative mutants, survivin-specific cytolytic T cells, a non-phosphorylatable survivin mutant Thr^34^ → Ala (T34A), and most recently, binding interface mimetics (Olie *et al*, 2000; Andersen *et al*, 2001; Grossman *et al*, 2001; Kanwar *et al*, 2001; Mesri *et al*, 2001; Wall *et al*, 2003; Plescia *et al*, 2005).

In the present study, we investigate whether extracellular compartmentalisation of survivin participates in the cytoprotection, tumourigenesis, and enhanced metastasis encountered in the tumour microenvironment. We have identified a novel pool of survivin, localised extracellularly, that is readily taken up by cancer cells but not by normal stromal cells. Furthermore, cancer cells that absorb survivin show enhanced growth patterns and are more resistant to genotoxic stress than controls containing only endogenous levels of survivin. Further investigations to define uptake-induced phenotypes of the apoptosis inducing non-phosphorylatable survivin-mutant (T34A) have shown a caspase-dependent enhancement of chemotherapy-induced cell death, *in vitro*.

## Materials and methods

### Cell lines and cultures

Cervical carcinoma (HeLa and HeLa-S), osteosarcoma (U2OS and SaOS2), pancreatic carcinoma (Panc1, Capan1, and Capan2), prostate carcinoma (PC3, DU-145, and RWPE-2), and breast carcinoma (MCF-7, HCC 1806) cell lines were obtained from the American Type Culture Collection (ATCC) as were the human normal prostate stromal cells (PrSC). The acute monocytic leukaemia cell lines (MOLM-14 and MV4-11) were a kind gift from CS Chen (Loma Linda University). Normal prostate epithelial cells (PrEC) were obtained from Clonetics-BioWhittaker (Walkersville, MD, USA) and cultured in PrEMB medium (Clonetics-BioWhittaker). The normal human bone marrow stromal (HBMS) cells were a generous gift of Kimberly Payne (Loma Linda University). Stromal cells were isolated and propagated as previously described (Hao *et al*, 1995) from adult human bone marrow that was purchased from Poietics Cell Systems (Lonza Walkersville Inc., Gaithersburg, MD, USA). Human peripheral blood mononuclear cells were isolated from a peripheral blood draw using Ficoll-Hypaque (GE Healthcare Bio-Sciences Corp., Piscataway, NJ, USA) as has been previously described (Wang *et al*, 1984). The use of all human tissues was reviewed and approved by the institutional review board at Loma Linda University with informed written consent obtained from each subject. Cells were maintained as defined in DMEM, McCoy's, RPMI, or IMEM (ATCC) supplemented with 100 U of penicillin, 100 *μ*g ml^−1^ streptomycin, 300 *μ*g of L-glutamine, and 10–20% heat inactivated fetal bovine serum (FBS; ATCC). Cells were grown at 37°C in a humidified atmosphere of 95% air, 5% CO_2_ until 60% confluent and supernatants were removed. New medium was given and cells were grown to 90% confluent, after which supernatants were collected, centrifuged for 10 min at 2500 r.p.m., and stored at −20°C until use.

### Expression plasmid and generation of stable cell lines

The detailed procedure for cloning and propagation has been described previously (Ogawa *et al*, 2002; Shi *et al*, 2003). In brief, recombinant retroviruses expressing a bicistronic messenger RNA containing open-reading frames of Flag-HA (haemagglutinin)-tagged human survivin or human T34A survivin and interleukin-2 receptor (IL-2R)-*α* were constructed and transduced into HeLa cells. The infected HeLa cells were sorted by anti-IL-2R monoclonal antibody (mAb) conjugated with magnetic beads, and the resulting Flag-HA-survivin or Flag-HA-T34A survivin stable cell lines propagated as suspension cultures. The expression level of both the wild-type (WT) and mutant (T34A) survivin was evaluated by western analysis and immunohistochemistry with anti-Flag and HA antibodies (Santa Cruz Biotechnology Inc., Santa Cruz, CA, USA).

### Survivin depletion

Conditioned medium (CM) from stable survivin-expressing HeLa cells contains survivin that has a Flag-HA tag as well as normal endogenous survivin. To deplete the medium of survivin, we added anti-Flag beads (20 *μ*l ml^−1^) to the medium and rotated overnight at 4°C. The medium was centrifuged at 2000 r.p.m. for 5 min to pellet the beads. The supernatant was collected and 100 *μ*l set aside for analysis by ELISA. The pelleted beads were collected and put in at −20°C. The remaining medium was treated with anti-HA beads (20 *μ*l ml^−1^) in a similar manner to remove the remaining tagged survivin. To deplete the endogenous survivin, we added anti-survivin polyclonal antibodies (10 *μ*l ml^−1^) and anti-rabbit beads (20 *μ*l ml^−1^) to the medium and rotated overnight at 4°C. The 100 *μ*l aliquots at each stage were used in an ELISA to confirm the depletion. The beads collected at each step were incubated in 40 *μ*l sample loading buffer at 100°C for 4 min, then centrifuged at 2000 r.p.m. for 5 min. A western blot of the supernatant was used to further confirm depletion.

### Quantitation of survivin from CM (cell culture supernatants)

Conditioned medium was collected from 90% confluent cell cultures with cell viability assessed by Trypan blue exclusion (Sigma-Aldrich, St Louis, MO, USA). One-half of the medium was concentrated 4 × using a speed-vac (Savant OligoPrep 120) after which unconcentrated and concentrated samples were assayed for the presence of survivin using the Quantikine Human Survivin immunoassay kit (R&D Systems Inc., Minneapolis, MN, USA), according to the manufacturer's instructions. To quantify representative survivin protein from cultures experiencing cell death, we grew HeLa cells to 90% confluence as previously described after which 0.1, 1.0, and 10% of these cells were lysed and their protein used to ‘spike’ control medium for ELISA.

### Immunofluorescence localisation of protein

Cells were plated in six-well plates on 22 × 22 mm coverslips and cultured with 2 ml of control or CM (Surv-WT-CM, Surv-T34A-CM). Wells were washed with PBS and fixed in 4% paraformaldehyde for 30 min at room temperature. Cells were permeabilised with 0.5% NP-40 in PBS for 15 min, blocked with 5% FBS in 0.01% NP-40/PBS for 30 min, incubated for 1 h with 1 : 500 dilution of anti-Flag/HA mAbs (Sigma-Aldrich). Cells were incubated with Alexa Fluor-555-conjugated rabbit anti-mouse IgG secondary antibody (Invitrogen/Molecular Probes, Eugene, OR, USA), mounted with Vectashield mounting media containing DAPI (Vector Laboratories, Burlingame, CA, USA) and observed ( × 1000) under an Olympus BX50 fluorescent microscope (Olympus America Inc., Center Valley, PA, USA).

### Image acquisition using laser-scanning confocal microscopy

Sampled sections were imaged and analysed with an Olympus FV 1000 laser-scanning confocal imaging system mounted onto an Olympus IX81 microscope (Olympus America Inc.). Microscopic data were acquired with a 60 × oil immersion objective lens. In each section, confocal stack images from representative fields of view per section were captured. The distance between each focal plane was 1 *μ*m.

### Protein expression and purification

Recombinant WT or mutated (T34A) proteins were expressed as glutathione-*S*-transferase (GST) fusion proteins in the *Escherichia coli* BL21-CodonPlus-RIL (Stratagene, La Jolla, CA, USA) strain with induction in 0.2 mM isopropyl-*β*-D-thiogalactopyranoside (IPTG) for 5 h at 30°C. BIRC5 (survivin) was purchased from Abnova (Walnut, CA, USA). The cells were harvested by centrifugation at 6000 **g** for 20 min, suspended in 50 mM Tris (pH 7.4), 150 mM NaCl, 5 mM MgCl_2_, and 1 mM dithiothreitol, and lysed by sonication. After centrifugation at 15 000 **g** for 30 min, we mixed the soluble fractions with glutathione-agarose beads (Sigma-Aldrich) and incubated for 1 h at 4°C. After centrifugation at 1000 **g** for 1 min, we washed beads containing proteins three times in 50 mM Tris (pH 7.4), 500 mM NaCl, 5 mM MgCl_2_, and 1 mM dithiothreitol and further purified by chromatography over Econo-Column (Bio-Rad, Hercules, CA, USA) after overnight thrombin (Sigma-Aldrich) treatment to release the GST frame. The protein concentrations were measured using a protein assay reagent (Pierce, Rockford, IL, USA) with BSA as standard. Recombinant transferrin protein was purchased from Sigma-Aldrich. The expression vector PRSET-CTB contained a gene encoding the entire cholera toxin B subunit (CTB) protein (11.6 kDa), driven by the T7 bacteriophage promoter region and containing an oligonucleotide encoding 6 histidines immediately upstream of the CTB gene for nickel column isolation of the recombinant protein was built as described (Carter *et al*, 2006). Briefly, CTB protein synthesis was stimulated by addition of 90 mg IPTG to the bacterial culture for 6 h at 37°C. Cells were harvested by centrifugation with the pellet resuspended in 10 mM HEPES buffer (pH 7.5), containing 100 mM imidazole. The cells were disrupted by sonication at 3 × 10 s bursts at 10 W, with a Sonic 60 Dismembrator. Cholera toxin B subunit protein was isolated from the homogenate by a Maxwell Model 16 robotic protein purification system (Promega Inc., Madison, WI, USA), according to the protein isolation protocol provided by the manufacturer. Purity of the isolated CTB protein was determined by electrophoretic mobility measurement in a 12% polyacrylamide gel. Imidazole was removed from the protein mixture by dialysis of the preparation against 10 mM HEPES buffer (pH 7.5), for 4 h at 4°C.

### IR-Dye labelling of recombinant proteins and in-cell western blotting

IR-Dye labelling was performed according to the manufacturer's instructions (LI-COR Biosciences, Lincoln, NE, USA). Briefly, 100 *μ*g of transferrin, CTB, and 10 *μ*g of BIRC5 was labelled at 20°C for 2 h. Labelled protein was column purified as described by the manufacturer. HeLa cells (5 × 10^3^ per well) were plated in 96-well plates. After overnight culture, we added labelled BIRC5, transferrin, and CTB proteins at doses of 1 *μ*g, 0.01 *μ*g, and 0.001 *μ*g in duplicate wells. After overnight culture, we washed cells vigorously 3–4 times with PBS to remove all remaining unattached recombinant protein. Cells were fixed with 4% paraformaldehyde and an in-cell western blot was performed using survivin polyclonal antibody (Novus, Littleton, CO, USA) according to the manufacturer's instructions. The 96-well plate was then scanned immediately in both 700 and 800 nm channels. Relative fluorescent intensity was determined using our Odyssey Infrared Imaging system and software.

### Colony formation assay

The cells were plated in 10 cm dishes at a density of 100 000 cells per well, incubated at 37°C overnight, and then were treated with the indicated control, Surv-WT-CM, and Surv-T34A-CM for up to 48 h. The cells were fixed with methanol and stained with crystal violet.

### Cell-cycle synchronisation

HeLa-S cells were G_1_-arrested for 16 h using mimosine as previously described (Li *et al*, 1999). Cells were then released and treated with either Surv-WT-CM or control medium and then harvested after 0, 3, 6, 9, and 12 h. Cells were analysed for DNA content by propidium iodide staining and flow cytometry (Becton Dickinson, San Jose, CA, USA).

### Apoptosis and cell-cycle analysis

Subconfluent cultures of the various cell lines were incubated with vehicle (DMSO), taxol (4 *μ*M; Sigma-Aldrich), 5-flurouracil (5-FU, 200 nM; Sigma-Aldrich), adriamycin (Ad, 100 nM, Sigma-Aldrich), cisplatin (CDPD, 2 *μ*M; Sigma-Aldrich) or exposed to ultraviolet B (UVB) irradiation at 50 J m^−2^ with or without the presence of WT-CM, T34A-CM, or control for 24 and 48 h at 37°C. Cells were harvested, prepared, and analysed for DNA content as described previously (Li *et al*, 1999). DNA content was analysed using a Becton Dickinson FACScan flow cytometer (Becton Dickinson). The distribution of cells in the different phases of the cell cycle was analysed from DNA histograms using BD CellQuest software (Becton Dickinson).

### AlamarBlue (AB) assay for cell proliferation

Initial experiments were carried out to follow per cent AB (%AB) reduction over time. The aim was to determine the optimal seeding density and culture period. HeLa cells, trypsinised from subconfluent cultures as described earlier, were suspended in culture medium containing 1% FBS, T34A-CM, and WT-CM and then seeded into duplicate wells of a 96-well plate (200 ml well) at concentrations of 1.5 × 10^4^–1 × 10^6^ cells per ml at standard culture conditions of 5% CO_2_ in air at 37°C. After an initial 4 h period to allow for cell attachment, we added AB (BioSource, Camarillo, CA, USA) directly into culture medium at a final concentration of 10% and the plate was returned to the incubator. Optical density of the plate was measured at 540 and 630 nm with a standard spectrophotometer at 1, 3, 6, 12, 24, 48, and 72 h after adding AB. Because the culture medium was not changed during this period, the calculated %AB reduction is a cumulative value. As a negative control, AB was added to medium without cells.

### Caspase activity assay

Caspase activity was quantitated using the ApoAlert Caspase Assay Plates (Clontech, Mountain View, CA, USA). Fluorogenic substrates specific for caspase-3, caspase-8, caspase-9, and caspase-2 were immobilised in the wells of a 96-well plate. Fluorescence was detected using a standard fluorescence plate reader in the presence or absence of the specific caspase inhibitor.

### Western blot analysis

Cells were solubilised, proteins (20–40 *μ*g) separated using 12% Bis-Tris polyacrylamide gels, proteins transferred onto polyvinylidene difluoride membranes (Millipore, Temecula, CA, USA) and probed using the following antibodies: mouse monoclonal anti-Flag and anti-HA (Sigma-Aldrich), rabbit polyclonal anti-survivin (Novus), rabbit polyclonal antibodies to caspase-7, caspase-9, PARP and GAPDH (Cell Signaling Technologies, Beverly, MA, USA), mouse mAbs to *β*-actin (Abcam, Cambridge, MA, USA), and mouse mAb to caspase-2 (Cell Signaling Technologies). Secondary antibodies (IR-Dye-conjugated) were goat anti-rabbit and goat anti-mouse immunoglobulin (LI-COR Biosciences). Immunoreactive bands were detected using the Odyssey imaging system (LI-COR Biosciences) and quantified using ImageQuant software (Amersham Biosciences, Piscataway, NJ, USA). Protein quantifications presented in this report were normalised with respect to *β*-actin or GAPDH.

### Mitochondrial permeability detection

Mitochondrial depolarisation is detected by a unique fluorescent cationic dye 5,5′,6,6′-tetrachloro-1,1′,3,3′-tetraethyl-benzamidazolocarbocyanin iodide, also known as JC-1 (Invitrogen/Molecular Probes). Cells were collected and washed with PBS and prepared for the analysis according to the manufacturer's instructions.

### Collagen cell invasion assay

Cellular invasion was studied using the QCM 96-well Fluorometric Collagen-based Cell Invasion Assay from Chemicon International (Millipore) according to the manufacturer's instructions. Briefly, single-cell suspensions were obtained by trypsinisation of monolayer cultures of HeLa cells. Cell counts and viability were performed using Trypan blue staining after overnight serum starvation. Cells (25 000 cells per well) were plated on 8 *μ*m pore size collagen-coated inserts in 96-well plates. Cells were grown for 24 h with control media, Surv-WT-CM, Surv-T34A-CM, medium that was depleted of survivin introduced into the lower chamber or medium taken from cells treated with cytochalasin D, a known inhibitor of exocytosis that works by specifically disrupting microfilaments. Survivin was depleted using one round of anti-Flag antibody, one round of anti-HA antibody, and either 1 (depleted 1 × ) or 3 (depleted 3 × ) rounds of anti-survivin antibody. A control, termed blank, was performed using only cell detachment buffer, lysis buffer, and CyQuant dye without cells. Invasive cells, migrating through the polymerised collagen layer, were dissociated using cell detachment buffer as described by the manufacturer and lysed. Fluorescence emission (CyQuant GR dye) was assessed by a fluorescent plate reader (Molecular Dynamics, Sunnyvale, CA, USA).

### Statistical analysis

Statistical analysis was performed using a two-way analysis of variance with the aid of JMP statistical software (Cary, NC, USA). A paired *t*-test was used for group analysis. Caspase-2, caspase-7, and caspase-9 densitometric analysis was conducted using our AlphaImager EC (Alpha Innotech, San Leandro, CA, USA). Density of individual bands was divided by GAPDH as the internal control with each timed sample then divided by the particular time period control. As 6 h staurosporine did not have a 6 h control, these were also controlled against the 24 h control samples.

## Results

### Stable expression of wild-type and mutant T34A survivin leads to survivin secretion

We and others (Bokarewa *et al*, 2005; Mera *et al*, 2008) recently found and reported a form of survivin that is found in the extracellular space. Using a commercially available survivin ELISA, we were able to quantitate picogram amounts of survivin from CM taken from tumour cell lines representative of the most common cancer types, including pancreatic, breast, prostate, cervical, sarcoma, and acute monocytic leukaemia (Table 1). To determine whether this extracellular form of survivin was merely released as a result of an excretory process, the result of cellular necrosis, or whether its function was secretory and would be important in the context of the tumour microenvironment, that is absorbed into cells, we needed to construct a form of survivin different from the endogenous form. We therefore generated stable HeLa cells expressing human survivin (Surv-WT) tagged with both the Flag and HA epitopes at their N terminus. Immunohistochemistry (Supplementary Figure 1A) as well as western blotting (Supplementary Figure 1C) using antibodies to either HA or Flag revealed the survivin fusion protein.

The Surv-T34A mutant has shown promise in initiating the mitochondrial apoptotic pathway (Mesri *et al*, 2001) and if also detected extracellularly could provide a form of survivin suitable for therapeutic manipulation. We therefore generated stable HeLa cells expressing the non-phosphorylatable Surv-T34A mutant. Immunohistochemistry (Supplementary Figure 1B) and western blotting (Supplementary Figure 1C) revealed the HA-Flag-survivin fusion protein. The generation of this cell line took many attempts, and many months, as the Surv-T34A mutant was very toxic to the HeLa cells. Further analysis of allowing mutations has not yet taken place.

**Figure 1:**
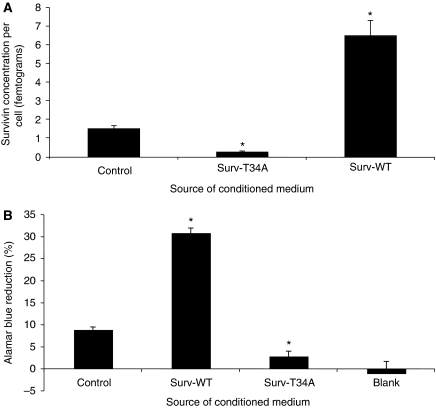
Comparison of extracellular survivin protein concentrations and cellular metabolism of the cervical carcinoma HeLa cells that were stable transfectants of the survivin-WT (Surv-WT) or mutant survivin T34A (Surv-T34A). (**A**) ELISA-defined survivin concentrations were measured in medium taken from the different stable HeLa cells lines as described in Materials and methods section. For each cell line, the survivin protein concentration was divided by the number of cells defined by Trypan blue exclusion at the time of medium collection. (**B**) Alamar Blue fluorimetry was conducted in the same three cell lines to determine if the stable expression of survivin or its mutant would affect the cell growth and metabolism. Measurements were also conducted in medium containing no cells (blank), which would represent no growth or metabolism. Data are the mean±s.e. of three independent experiments (^*^P<0.001) as compared to the control.

Conditioned medium taken from these stable Surv-WT or Surv-T34A HeLa cells showed picogram amounts of survivin by ELISA (Table 1). Interestingly, it was quite apparent that accumulation of the T34A mutant survivin and that from the parent HeLa-S cells was limited in comparison to that which accumulated in the medium from the Surv-WT cells (Table 1). To evaluate this disparity, we evaluated survivin protein concentration, determined using ELISA, on a per cell basis. Dividing the measured amount of protein by the number of cells that were present provides evidence that the Surv-WT cells truly do release more protein into the CM than does the control cells or the Surv-T34A-producing cells (Figure 1A). AB staining was next used to determine if this phenomenon was the result of cellular division. As recorded in Figure 1B, AB reduction in the Surv-WT HeLa-S cells represented a state of enhanced cellular growth when compared to the controls or the Surv-T34A cells. Surv-T34A HeLa-S cell proliferation is significantly reduced when compared to the Surv-WT and control HeLa-S cells. Also, there was no significant difference in the level of cell proliferation recorded in the Surv-T34A-expressing cells when compared to medium where no cells were being grown (Figure 1B, blank), indicating that Surv-T34A may reduce the level of released survivin as a result of its stifling cell growth.

**Figure 2:**
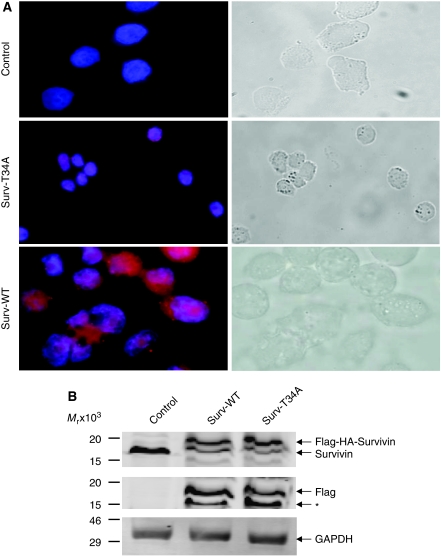
HeLa cells incubated in Surv-WT- or Surv-T34A-conditioned medium take up the Flag-HA-survivin fusion protein. (**A**) HeLa cells were cultured with control-, Surv-WT-, or Surv-T34A-conditioned medium for 24 h. Cells were fixed and stained with antibodies to HA, magnification × 1000. Insets are western blots taken from cell lysates of these cells probed with antibodies to HA. Phase-contrast panels show the T34A-associated shrunken phenotype and early apoptotic bodies in comparison with the control or WT-treated cells. (**B**) Western blot analysis using antibodies to both survivin and Flag shows the retarded ∼18 kDa band of the Flag-HA-survivin fusion protein. Control lysates only show the presence of the 16.5 kDa endogenous survivin. Molecular weight markers in kilodaltons (kDa) are shown on the left.

**Figure 3:**
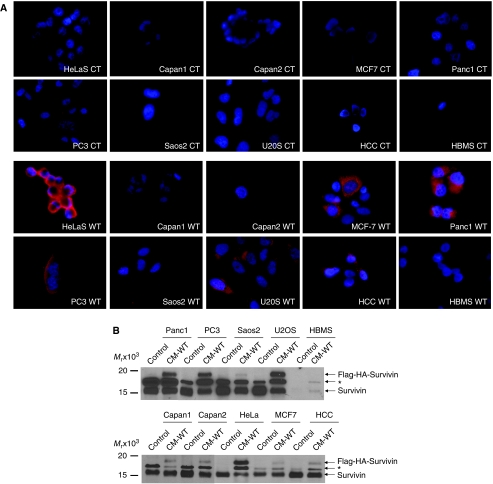
Cancer cells but not normal cells incubated in Surv-WT-conditioned medium take up the Flag-HA-survivin fusion protein. (**A**) HeLa, Capan1, Capan2, MCF-7, Panc1, PC3, SaOS2, U2OS, HCC, and HBMS cells were cultured with control- or Surv-WT-conditioned medium for 24 h. Cells were fixed and stained with antibodies to HA, magnification × 1000. (**B**) Western blot analysis using antibodies to survivin shows the retarded ∼18 kDa band of the Flag-HA-survivin fusion protein only in those lysates from the cells grown in conditioned medium. A third band, labelled ^*^, often appears after probing with the antibodies survivin whose identity is yet unknown to us. Molecular weight markers in kilodaltons (kDa) are shown on the left.

**Figure 4:**
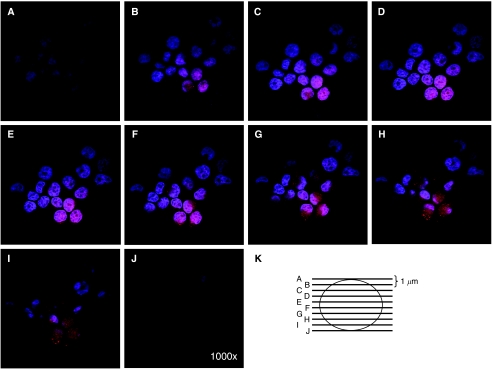
Confocal microscopy indicates that survivin protein is taken up by cancer cells and is not merely binding to the cell surface. Cells were fixed and stained with antibodies to HA, with pictures taken on a *z*-scale of 1 *μ*m, magnification × 1000. (**A–J**) are *z*-scale pictures taken at 1*μ*m intervals as represented in (**K**).

We next evaluated the functional importance of this extracellular survivin protein. HeLa cells were incubated for 24 h in CM taken from control, Surv-WT, or Surv-T34A stable cells. Immunohistochemical staining (Figure 2A) and western blotting (Figure 2B), using antibodies to HA, showed that the survivin fusion proteins are readily taken up. A retarded band of approximately 18 kDa appeared in the HeLa-S lysates of cells incubated in WT- and T34A-CM representing the Flag-HA-survivin fusion protein (Figure 2B), which was not observed in control conditioned medium grown HeLa cells. Interestingly, the cells that were incubated in Surv-T34A-CM took on a shrunken appearance and were fewer in number compared to the control medium-incubated cells (Figure 2A) whereas the Surv-WT, CM-incubated cells appeared to be healthier and in greater numbers. Phase-contrast microscopy allowed enhanced visualisation of this shrunken phenotype and the initiation of apoptotic bodies in these T34A-CM-treated cells. To ensure that the ability of cells to uptake survivin from the CM was a general occurrence and not specific for the HeLa cells, we also incubated Capan1, Capan2, MCF-7, Panc1, PC3, SaOS2, U2OS, HCC, and HBMS cells in HA-Flag-survivin-containing CM. All cancer cells were able to take up the survivin from the medium whereas the normal HBMS cells did not (Figure 3A). Western blots (Figure 3B) from these cells confirmed the uptake results as every cell type except HBMS, when incubated in Surv-WT-CM, expressed 18 kDa band representing the Flag-HA-survivin fusion protein. Previous work performed on neutrophils showed survivin not entering the cells but binding to the surface (Mera *et al*, 2008). It was therefore necessary to perform confocal microscopy to ensure survivin's relocalisation within the cells. Taking photographs at 1 *μ*m increments throughout the cell (shown schematically in Figure 4K) indicated that in these HeLa cells, survivin was not only inside the cells but also relocalised within the cytoplasm and to the nucleus (Figure 4).

### Relationship between survivin absorption, Surv-T34A-induced apoptosis, and Surv-WT-induced cell-cycle progression

**Figure 5:**
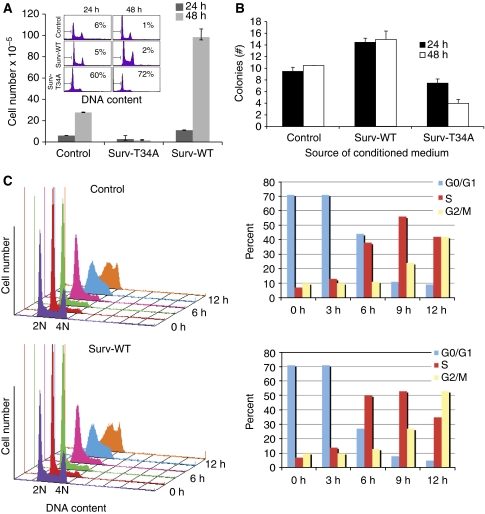
Surv-T34A-conditioned medium induces tumour cell apoptosis whereas Surv-WT-conditioned medium promotes cell growth. (**A**) Cervical carcinoma HeLa cells were grown in the presence of either Surv-T34A- or Surv-WT-conditioned medium for 24 and 48 h, harvested and analysed for DNA content by propidium iodide staining and flow cytometry (inset) or Trypan blue exclusion. Percentages of apoptotic cells with hypodiploid (sub-G_1_) DNA content are indicated per each condition tested. Data are representative of one of two or three independent experiments with comparable results. (**B**) HeLa cells, grown as described, were fixed with methanol and stained with crystal violet allowing visualisation of the enhanced cellular proliferative effects of Surv-WT or the killing/growth-repressive effects of Surv-T34A. The data presented are the mean colony formation efficiency±s.d. in three experiments. (**C**) Mimosine-synchronised HeLa cells were harvested at the indicated increasing time intervals after release and analysed for DNA content by propidium iodide staining and flow cytometry. A full colour version of this figure is available at the *British Journal of Cancer* online.

Consistent with the data presented in Figure 2A, HeLa cells grown in CM taken from stable Surv-T34A cells experienced enhanced numbers of hypodiploid cells, that is, sub-G_1_ DNA content or apoptotic cells (Figure 5A, inset) by DNA content analysis and flow cytometry. Furthermore, Trypan blue exclusion and colony formation assay evaluation of these same cells showed a time-dependent, marked reduction in live cell numbers and live cell colonies in the Surv-T34A-CM-grown HeLa cells compared with cells grown in control medium or the CM from the Surv-WT cells (Figures 5A and B and Supplementary Figure 2). This cell number reduced even further after 48 h as shown by the live cell colonies in the Surv-T34A-CM-grown HeLa cells, a finding that is consistent with what has been recorded by our own group and others (Mesri *et al*, 2001; O'Connor *et al*, 2002; Wall *et al*, 2003; Ogura *et al*, 2008) that the dominant-negative survivin mutant T34A induces as caspase-dependent apoptosis. In contrast, Trypan blue exclusion and colony formation assay evaluation of Surv-WT-CM-grown cells showed a time-dependent, marked increase in live cell numbers (four times) and live cell colonies (approximately two times), in comparison with cells grown in control medium or the CM from the Surv-T34A cells (Figures 5A and B and Supplementary Figure 2).

To further evaluate the cell-cycle progression effects observed in the Surv-WT-CM-grown cells, we mimosine synchronised HeLa cells for 16 h, which effectively arrested cells in the G_0_/G_1_ phase of the cell cycle. Mimosine was then removed and the cells were incubated in the absence or presence of Surv-WT-CM. Cells were then harvested at 0, 3, 6, 9, and 12 h time points following CM addition and cells were analysed by DNA content analysis and flow cytometry. In comparison with cells grown in control medium, Surv-WT-CM-grown cells had a 10–12% increase in S-phase growth in cells harvested after 6 h and a 10% increase in the number of cells in G_2_/M by 12 h (Figure 5C). These findings are consistent with those published indicating that survivin displays both anti-apoptosis and promotion of mitosis in cancer cells (Li *et al*, 1998; O'Connor *et al*, 2000; Chao *et al*, 2007). We are further encouraged as our CM containing fusion proteins may prove useful for the future study of survivin trafficking and T34A therapy *in vivo*.

### Recombinant survivin protein lacks the function of survivin-containing CM

**Figure 6:**
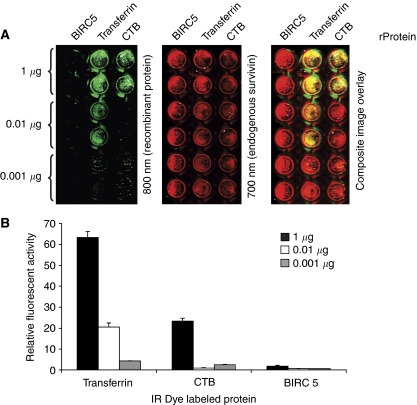
Recombinant survivin protein is unable to cross the HeLa cell membrane. (**A**) Detection of cellular recombinant proteins. These images show a portion of a 96-well plate. The right panel is a composite image showing the fluorescence in both the 700- and 800-nm detection channels. Duplicate rows of microplate wells loaded with 0.001, 0.01, or 1.0 *μ*g of protein are shown. The middle panel shows detection of endogenous survivin protein, which was used as a loading control. The left image shows detection of increasing amounts of recombinant survivin (BIRC5), transferrin, and CTB. (**B**) Quantification of fluorescence. Recombinant protein signal has been normalised using the endogenous survivin protein signal from each well to correct for well-to-well variation in cell number. Recombinant protein (rProtein).

Recombinant Surv-WT and Surv-T34A proteins were incapable of affecting cell growth or apoptosis in our hands (Supplementary Figure 3). It is uncertain whether bacterial-derived survivin proteins are folded or modified properly for recognition by cellular mechanisms of uptake. To test this hypothesis further, we evaluated recombinant survivin (BIRC5), transferrin, and CTB proteins for cellular uptake using in-cell western assay methods (Egorina *et al*, 2005). HeLa cells were incubated with increasing concentrations of labelled recombinant protein, and the amount of protein endocytosed after a 12 h incubation was measured as shown in Figure 6. The amount of transferrin and CTB proteins endocytosed increased in a dose-dependent manner. However, survivin recombinant protein (BIRC5) at the same concentrations was not endocytosed (Figure 6). Transferrin (Sandvig and van Deurs, 1990) and CTB (Gizurarson *et al*, 1992; Frey *et al*, 1996; Hansen *et al*, 2005) recombinant proteins were previously described as substrates for endocytic uptake and were used as positive controls.

### Characterisation of tumour cell apoptosis induced by Surv-T34A *in vitro*

**Figure 7:**
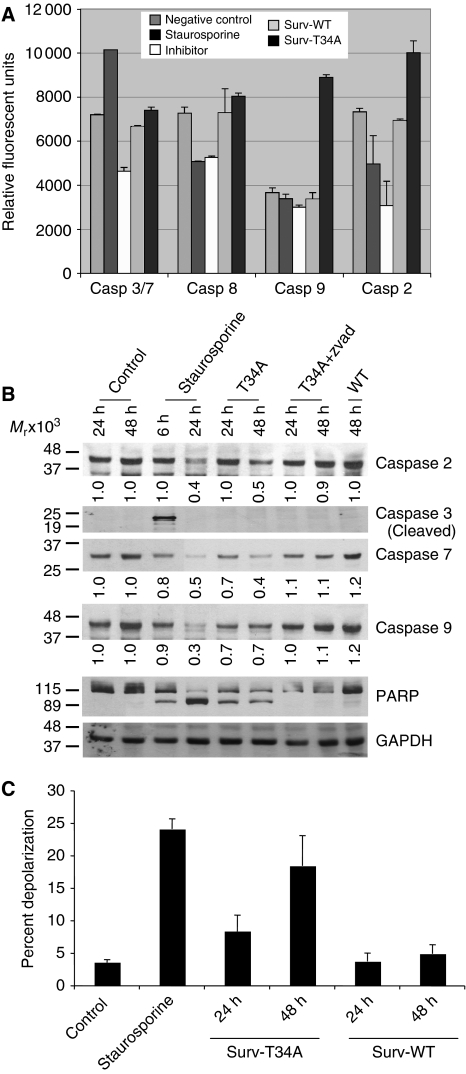
Conditioned medium containing survivin affects the intrinsic apoptotic pathway. (**A**) Caspase catalytic activity. Aliquots of HeLa cells grown in the presence of either Surv-T34A- or Surv-WT-conditioned medium were assayed for caspase-3/7, caspase-8, caspase-9, and caspase-2 activity by Ac-DEVD-AMC, IETD-AMC, LEHD-AMC, and VDVAD-AMC hydrolysis, respectively, in the presence or absence of the caspase-3 inhibitor, DEVD-CHO; the caspase-8 inhibitor, IETD-fmk; the caspase-9 inhibitor, LEHD-fmk; or the caspase-2 inhibitor, VDVAD-fmk. Inhibitor (INH), staurosporine (STR). Data are the mean±s.e. of two independent experiments. (**B**) Caspase activation. Detergent-solubilised extracts of HeLa cells grown in Surv-WT- or Surv-T34A-conditioned medium were analysed at the indicated time intervals for reactivity with antibodies for caspase-2, caspase-3, caspase-7, caspase-9, PARP, and GAPDH (loading control) by western blotting. Molecular weight (*M*_r_) markers in kilodaltons are shown on the left. Densitometric analysis was conducted on caspase-2, caspase-7, and caspase-9 with their densities as compared to GAPDH located below each blot. (**C**) Mitochondrial depolarisation. The experimental conditions are the same as in **B**, except that HeLa cells grown in Surv-WT- or Surv-T34A-conditioned medium were analysed at the indicated time intervals using the mitochondrial membrane potential monitoring agent JC-1. A reduction in Δ*ψ*_m_ results in diffusion of the dye from the mitochondria and a subsequent reduction in the mean red fluorescent intensity, which is quantifiable using flow cytometry. Staurosporine treatment for 6 h is used as a positive control. Data are the mean±s.e. of two independent experiments.

We have shown in Figures 2A and 5A that incubating HeLa cells with Surv-T34A-CM resulted in apoptosis. Cytofluorometric quantification results showed that in comparison with control media or Surv-WT-CM treatment, Surv-T34A treatments induced robust results within 24 to 48 h. Previous studies performed, using an adenovirus-encoding T34A mutant, resulted in apoptosis that was associated with the mitochondrial release of cytochrome *c*, cleavage of caspase-9, and the processing of caspase-3 (Mesri *et al*, 2001). Further analysis of Surv-T34A-CM-induced apoptosis, using a commercially available caspase activity assay, revealed that Surv-T34A induced cleavage of caspase-9 and caspase-2 without cleaving the substrates specific for caspase-3 or caspase-8 (Figure 7A). The antibiotic and protein kinase inhibitor, staurosporine, was used as a positive control for caspase-3 activity. Surv-WT-CM had no effect on the caspase activity under evaluation. In addition, western blotting provided biochemical data that supported studies recorded above for caspase-2, caspase-3, and caspase-9. Using antibodies that were specific for the full-length, non-cleaved forms of caspase-2, caspase-7, and caspase-9 and the cleaved form of caspase-3 protein and staurosporine again as a positive control, we found that Surv-T34A induced the parallel time-dependent reduction in caspase-2, caspase-7, caspase-9, and cleavage of the DNA repair protein PARP (Figure 7B). In all cases the pan-caspase inhibitor Z-VAD-FMK was able to inhibit the activity of Surv-T34A. In contrast, Surv-WT again had no observable effects on caspase cleavage when cells were treated with it alone for up to 48 h. Caspase-3 was robustly cleaved after 6 h of staurosporine treatment. However, this cleavage product was absent after 24 h indicating a complete cleavage and degradation of caspase-3. Concomitant with this cleavage was the significant level of PARP cleavage recorded at this 24 h time point.

### Loss of mitochondrial membrane potential after treatment with Surv-T34A CM

Mitochondrial membrane potential (Δ*ψ*_m_), a phenomenon readily measured using the mitochondrial dye JC-1 and fluorescence-activated cell sorting analysis (Salvioli *et al*, 1997), was evaluated for the possibility that Surv-T34A was stimulating apoptosis by inducing depolarisation within the mitochondria. Under normal circumstances, JC-1 accumulates in the inner mitochondrial membrane in which it oligomerises and fluoresces red. A reduction in Δ*ψ*_m_ results in diffusion of the dye from the mitochondria and a subsequent reduction in the mean red fluorescent intensity. As expected, control HeLa cells showed a high mean red fluorescence (set to ∼96%) after staining with JC-1 (Figure 7C). After treatment with staurosporine, we found that the mean red fluorescence of the mitochondria dropped rapidly within 6 h, indicating that the Δ*ψ*_m_ had collapsed (Figure 7C). HeLa cells treated with Surv-T34A, but not Surv-WT, showed a time-dependent reduction in the mean red fluorescence intensity. However, in repeated experiments this was always slower than that seen for staurosporine (Figure 7C). This may indicate that mitochondrial depolarisation due to Surv-T34A is a secondary event and not the primary effect of Surv-T34A-induced killing or that mitochondrial depolarisation is one of many factors involved in Surv-T34A-induced killing.

### Effect of Surv-T34A and Surv-WT and UV radiation on tumour cell apoptosis

**Figure 8:**
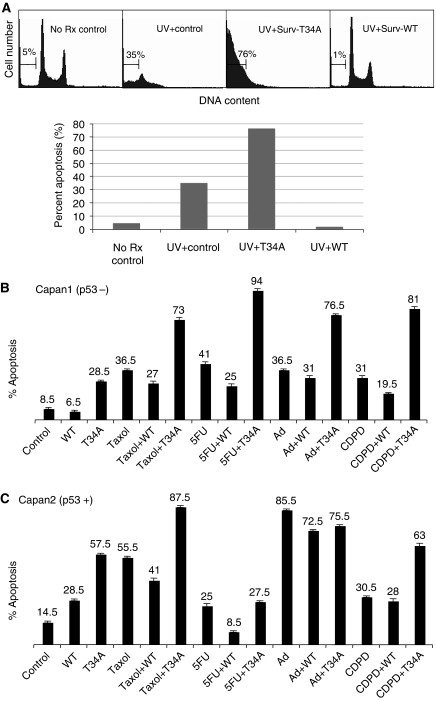
Effect of Surv-WT and Surv-T34A and UV (**A**) or chemotherapeutic drugs (**B** and **C**) on tumour cell apoptosis. Aliquots of HeLa cells were treated with UV (50 J m^−2^) (**A**) or Capan1 (**B**) and Capan2 (**C**) cells were treated with Adriamycin (Ad; 100 nM), 5-fluorouracil (5-FU; 200 nM), taxol (4 *μ*M), or cisplatin (CDPD; 2 *μ*M) in the presence or absence of Surv-WT- or Surv-T34A-conditioned medium. Cells were harvested at 48 h and apoptosis was determined by DNA content analysis and flow cytometry. The time point of combination treatments is 48 h after the addition of the drug following a 24-h pretreatment in the conditioned medium. Data are representative of one of two independent experiments with comparable results.

Incubation of HeLa cells with UV radiation resulted in enhanced numbers of hypodiploid cells, which are sub-G_1_ DNA content or apoptotic cells (Figure 8A) by DNA content analysis and flow cytometry. In comparison to control cells that were set at ∼5%, UV radiation treatment resulted in 35% of the cells exhibiting a sub-G_1_ DNA content. However, cells grown for 24 h in Surv-WT-CM before UV treatment were fully protected from the effects of the UV energy. In turn, Surv-T34A enhanced the killing effects of the UV and resulted in 75% of the cells exhibiting a sub-G_1_ content (Figure 8A).

### Effect of Surv-T34A and Surv-WT and chemotherapeutic drugs on tumour cell apoptosis

To establish that the effects recorded in HeLa cells were not specific for this cell line alone or for UV radiation, we selected human pancreatic adenocarcinoma (Capan1 and Capan2) cell lines to study drug sensitivity and responsiveness in the presence or absence of Surv-WT- and Surv-T34A-CM. Effects of taxol, 5-FU, Ad, and CDPD were studied. Capan1 cells exhibited an enhanced susceptibility to taxol, 5-FU, Ad, CDPD-induced cell death after the addition of Surv-T34A-CM, whereas Surv-WT-CM-incubated cells were protected from taxol, 5-FU, and CDPD-induced cytotoxicity (Figure 8B). Only a modest protection was recorded against Ad. Interestingly, Surv-WT-CM treatment protected Capan2 cells from the cytotoxic effects of 5-FU and again only a modest protection was recorded against taxol, Ad, and CDPD. Treatment of Capan2 cells with Surv-T34A-CM was as effective as taxol, more effective than CDPD and 5-FU and less effective as Ad alone. Surv-T34A when combined with taxol showed an additional cytotoxicity that may be quantitatively additive at best. When combined with CDPD, Surv-T34A did not provide further enhancement to the killing effects over that of Surv-T34A alone, which was substantial, and when paired with 5-FU or Ad, the individual cytotoxic effects of Surv-T34A were reduced (Figure 8C).

### Survivin containing CM induces cancer cell invasion *in vitro*

**Figure 9:**
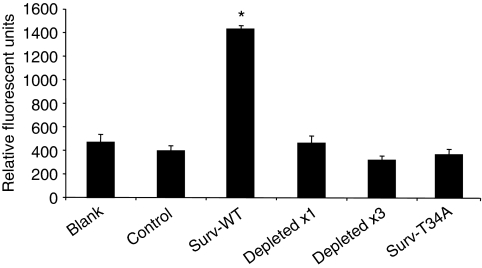
Effect of Surv-WT and Surv-T34A on tumour cell invasion. HeLa cells (1 × 10^5^ cells) were seeded into the upper well of the FIA chamber in 100 *μ*l culture medium. Cells were treated with the presence of Surv-WT- or Surv-T34A-conditioned medium, medium depleted of survivin using two rounds of fusion protein antibodies (anti-Flag and anti-HA) and one or three rounds of survivin antibodies in the lower chamber and incubated at 37°C in an atmosphere of 5% CO_2_. After 24 h of incubation, we lysed and quantitatively analysed fluorescence intensity of invasive cells that passed through the collagen layer onto the surface of the fluorescence-blocking membrane, giving relative fluorescent units (RFU). Data are the mean±s.d. of four independent experiments (^*^P<0.01).

To determine the function of secreted survivin in regulating cancer cell invasion through collagen, we plated HeLa cells on collagen-coated inserts in the presence of control, Surv-WT-, or Surv-T34A-CM. Cells were grown for 24 h, dissociated, lysed, and then evaluated for invasion by measuring the fluorescence emission (CyQuant GR dye). HeLa cells exhibited an average fourfold increase in cell invasion when grown with Surv-WT-CM in the lower chamber as compared to control medium (Figure 9). Surv-T34A-CM invasion levels were little changed from that of the control as were those cells that were treated with medium that had been depleted of survivin.

## Discussion

The growth and spread of cancer depends as much on the host response to the tumour as on the biological characteristics of the tumour itself. The IAP survivin has been shown aberrantly expressed in cancer but undetectable in normal differentiated adult tissue. It has been implicated in both control of apoptosis (Ambrosini *et al*, 1997; Adida *et al*, 1998) and regulation of cell division (Deveraux and Reed, 1999; Li *et al*, 1999; Gianani *et al*, 2001). Indeed, survivin expression has been shown to be cell-cycle regulated with its highest expression in G_2_/M phase, and it has been shown that much of its function comes from its subcellular localisation with residences in the cytosol, nucleus, and mitochondria (Li *et al*, 1999; Li, 2003). Recent reports on patients with rheumatoid arthritis have described a new survivin localisation and the possibility that it may also function in the extracellular space (Bokarewa *et al*, 2005; Mera *et al*, 2008). Moreover, in these patients, extracellular survivin was directly linked to an erosive course of joint disease and its origin was prescribed to peripheral blood leukocytes where its expression is constitutive. Furthermore, survivin was shown to bind extracellularly to neutrophils inducing the p38-MAPK-dependent expression of *α*- and *β*-integrins (Mera *et al*, 2008). Taken together, the finding of a new and physiologically functional pool of survivin provides new insight into the function of IAPs as well as other relevant tumour-associated proteins in the tumour microenvironment.

In this study we have shown that an extracellular form of survivin is detectable in CM taken from cancer cells. We also demonstrate that unlike survivin from the rheumatoid arthritis study, survivin from cancer cells is readily taken up by cancer cells rather than binding only to their surface (Mera *et al*, 2008). Finally, upon incubation in medium containing this extracellular pool of survivin, cancer cells responded physiologically, becoming more resistant to therapy, proliferating more rapidly, and having an increased metastatic potential. These findings lead us to believe that extracellular survivin may modulate, *in vivo*, the tumour microenvironment for the purpose of tumour evolution and these findings in part may be responsible for the observations that patients with a high expression of survivin protein display advanced disease, high-grade disease, abbreviated survival, resistance to therapy, and accelerated tumour recurrences (Andersen *et al*, 2007).

We have also shown that stable cells expressing the previously described (Mesri *et al*, 2001; O'Connor *et al*, 2002) pro-apoptotic survivin mutant Thr^34^ → Ala (Surv-T34A) release a form of survivin that is able to disrupt the cell cycle and cause a caspase-dependent, mitochondrial depolarisation-associated apoptosis. When combined with UV radiation or with the chemotherapeutic agents taxol, Ad, CDPD, or 5-FU, Surv-T34As pro-apoptotic ability was amplified. It was not surprising that these modalities were differentially enhanced or inhibited by Surv-T34A or Surv-WT because they have varied mechanisms of action. Surv-T34A was found to be as effective in enhancing therapy-induced cell killing as Surv-WT was in protecting against therapy-induced cell killing. These differential effects seemed not to follow the p53 status of the cell lines tested as Surv-WT was as effective at protecting those cells lacking the tumour suppressor and transcription factor as it was for those cells positive for p53. It has been shown that survivin gene transcription is repressed by WT p53 (Li, 2003) but little is known of p53's ability to modify the protein's function. A more careful evaluation of this finding is required.

The precise mechanism by which this survivin mutant causes apoptosis is still incompletely understood. The mutations that allowed for the generation of stable cells as well as their ability to induce apoptosis through the caspase-2- and caspase-9-dependent pathways are currently being evaluated in our laboratory. Importantly, a quantifiable highly stable extracellular form of survivin has been identified. This novel T34A survivin molecule is readily taken up by cancer cells after which characteristics of apoptotic cell death are measurable. Further, it potentiates the killing effects of other cancer therapeutics. It is our belief that this extracellular Surv-T34A molecule has significant potential as a novel therapeutic or as part of a novel therapeutic and possible immunotherapy regimen.

Tumour invasion and metastasis are very complex processes that are yet very poorly understood but account for 90% of all human cancer deaths (Sporn, 1996; Hanahan and Weinberg, 2000). The mechanism involves coupling and uncoupling of cells to their microenvironment, activation of extracellular proteases, and modulation in tethering proteins such as cadherins, *β*-catenin, and integrins (Hanahan and Weinberg, 2000). As survivin has already been shown to modulate the integrin proteins CD49d, CD11b, and CD11c on leukocytes (Mera *et al*, 2008), we hypothesised that it may also modulate the invasive capabilities of cancer cells. In our hands Surv-WT-CM stimulated a 3- to 4-fold increase in collagen invasion in comparison with the control medium alone or medium containing Surv-T34A. Medium depleted of survivin using antibodies first that recognised the fusion proteins Flag and HA and then one and three rounds of anti-survivin antibody depletion, completely inhibited survivin-induced collagen invasion thus placing survivin as a central player in this increased metastatic potential.

Stress-induced cancer cell death by means of apoptosis, necrosis, and autophagy occurs continually during tumour development and progression (Fonseca and Dranoff, 2008). What the ramifications of this to the tumour microenvironment is only beginning to be evaluated. It is believed that the capture of apoptotic cells serves as an immunoregulatory event that helps maintain tolerance whereas necrotic cells evoke an inflammatory cascade that mobilises effector responses. On the basis of our findings, we postulate that many of these released cancer antigens could in addition to modulating immune function prime the cells within the tumour microenvironment for the phenotypes associated with tumour invasion and metastasis that are listed above.

Survivin expression in CM was measured extracellularly using ELISA. It is not known whether extracellular survivin originates from dead cells or is the subject of active secretion. Comparing survivin quantity from 1 to 10% lysates to that of CM argues for active secretion rather than necrosis as SaOS2, Capan1, and HeLa-S-Surv-WT cells all have equal or more survivin protein but yet have no dead or dying cells as determined visually and by Trypan blue exclusion. Importantly, survivin found extracellularly creates a more erosive disease in patients with rheumatoid arthritis (Bokarewa *et al*, 2005) and in our cancer cell culture models, parameters that are considered clinically unfavourable in cancer patients: proliferative advantage, enhanced resistance to therapy, and enhanced metastatic potential. Investigating recombinant survivin's ability to recapitulate the effects recorded using CM-containing survivin proved unprofitable. From these experiments, we conclude that the survivin recombinant protein, which has been severed from its GST handle, is unable to induce similar phenotypes as the CM-containing survivin as it is unable to enter the cancer cells. Further studies using circular dichroism mass spectrometry will be required to compare these two forms of survivin to define differences in structure and or posttranslational modifications that may aid survivin in binding with its yet to be defined receptor or trafficking proteins.

A deeper understanding of the mechanisms underlying this extracellular pool of survivin, the possible ramifications for tumourigenesis, and the possibility of exploiting it for therapy are only a few of the many properties of this most fascinating of proteins that we are continuing to evaluate.

## Figures and Tables

**Table 1:** Quantitative analysis of survivin in conditioned medium of cancer cell lines by ELISA

**Cell type**	**Cell lines**	**pg ml^−1^**	**s.e.m.**	**Concentrated (pg ml^−1^)**	**s.e.m.**
Cervical cancer	HeLa	ND	—	330.4	11.4
	HeLa-S	94.0	42.1	746.1	1.4
	HeLa-S-Surv-WT	619.0	24.3	1158.3	32.1
	HeLa-S-Surv-T34A	35.2	0.01	81.9	0.01
	HeLa-S-survivinAntibody depletion	ND	—	ND	—
Pancreatic cancer	Capan1	246.9	13.6	1204.7	14.3
	Capan2	ND	—	26.1	8.6
	Panc1	20.4	42.9	174.7	47.1
Prostate cancer	PC3	73.3	32.9	888.3	5.0
	Du-145	136.9	37.4	771.9	24.0
	RWPE-2	29.0	16.0	337.6	7.0
Osteosarcoma	U2OS	ND	—	210.4	14.3
	SaOS2	405.4	50.0	858.3	50.7
Breast cancer	MCF-7	63.0	2.0	175.4	1.7
	HCC TN	ND	—	19.0	1.4
Acute monocytic leukaemia	MOLM-14	ND	—	273.3	23.3
	MV4-11	ND	—	347.6	15.9
Human bone marrow stroma	HBMS (normal)	ND	—	ND	—
Prostate epithelial cells	PrECs (normal)	ND	—	ND	—
Prostate stromal cells	PrSCs (normal)	ND	—	ND	—
Peripheral blood mononuclear cells	PBMCs (normal)	ND	—	ND	—
Media control	Media control	ND	—	ND	—
Spiked media control	0.1% lysed cells	ND	—	3.2	0.7
	1.0% lysed cells	77.6	1.1	121.9	2.0
	10% lysed cells	259.7	8.3	596.1	37.0

ND=none detected.
