# Supplementary material for: Extracellular, cell-permeable survivin inhibits apoptosis while promoting proliferative and metastatic potential
Source: Br J Cancer. 2009 Mar 17;100(7):1073–86. doi: 10.1038/sj.bjc.6604978 (PMC2669990; doi:10.1038/sj.bjc.6604978)
Supplement: Supplementary Figure 1–3 [file 6604978x1.ppt]

## Slide 1
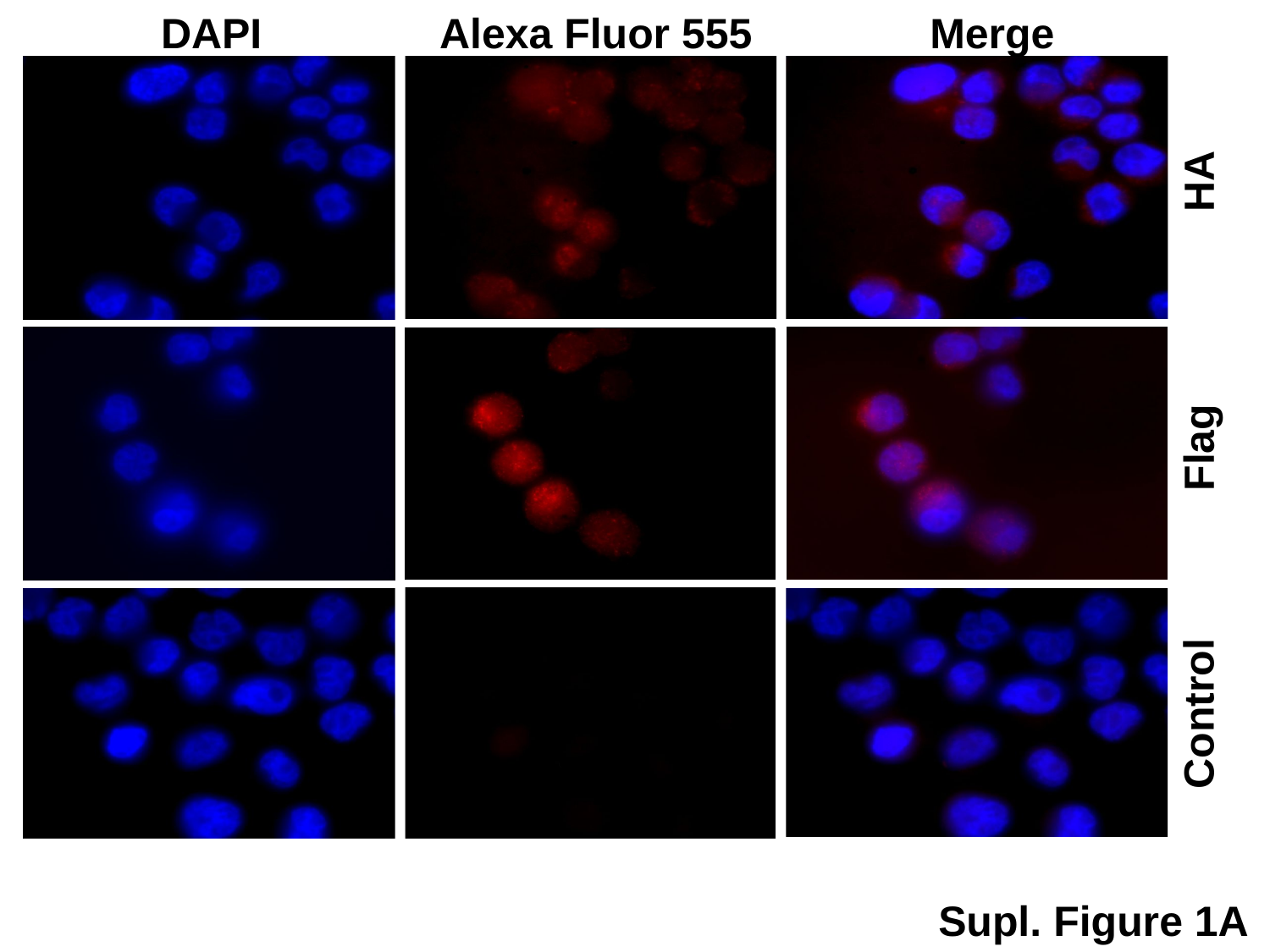

DAPI
Alexa Fluor 555
Merge
HA
Flag
Control
Supl. Figure 1A

## Slide 2
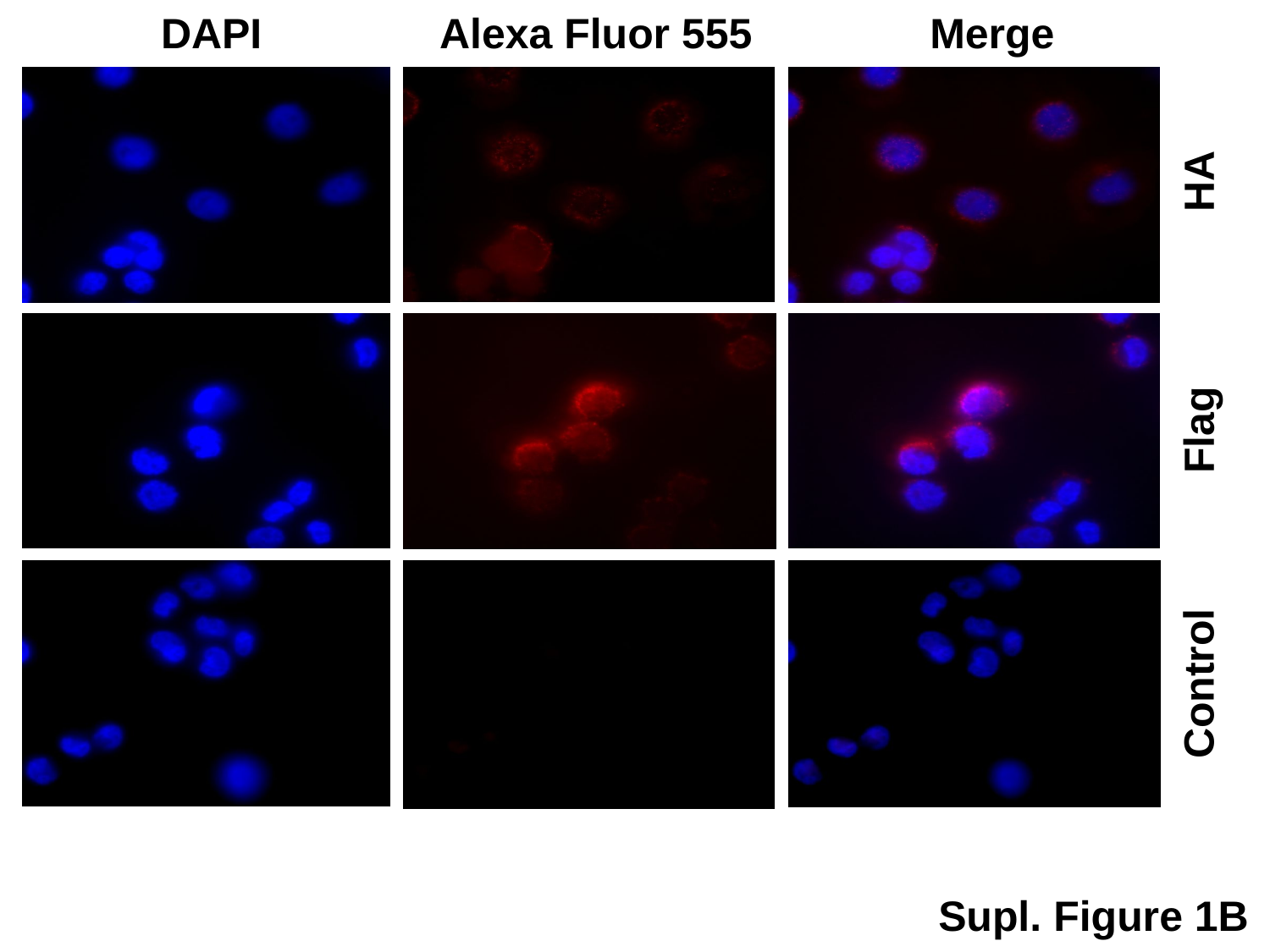

DAPI
Alexa Fluor 555
Merge
HA
Flag
Control
Supl. Figure 1B

## Slide 3
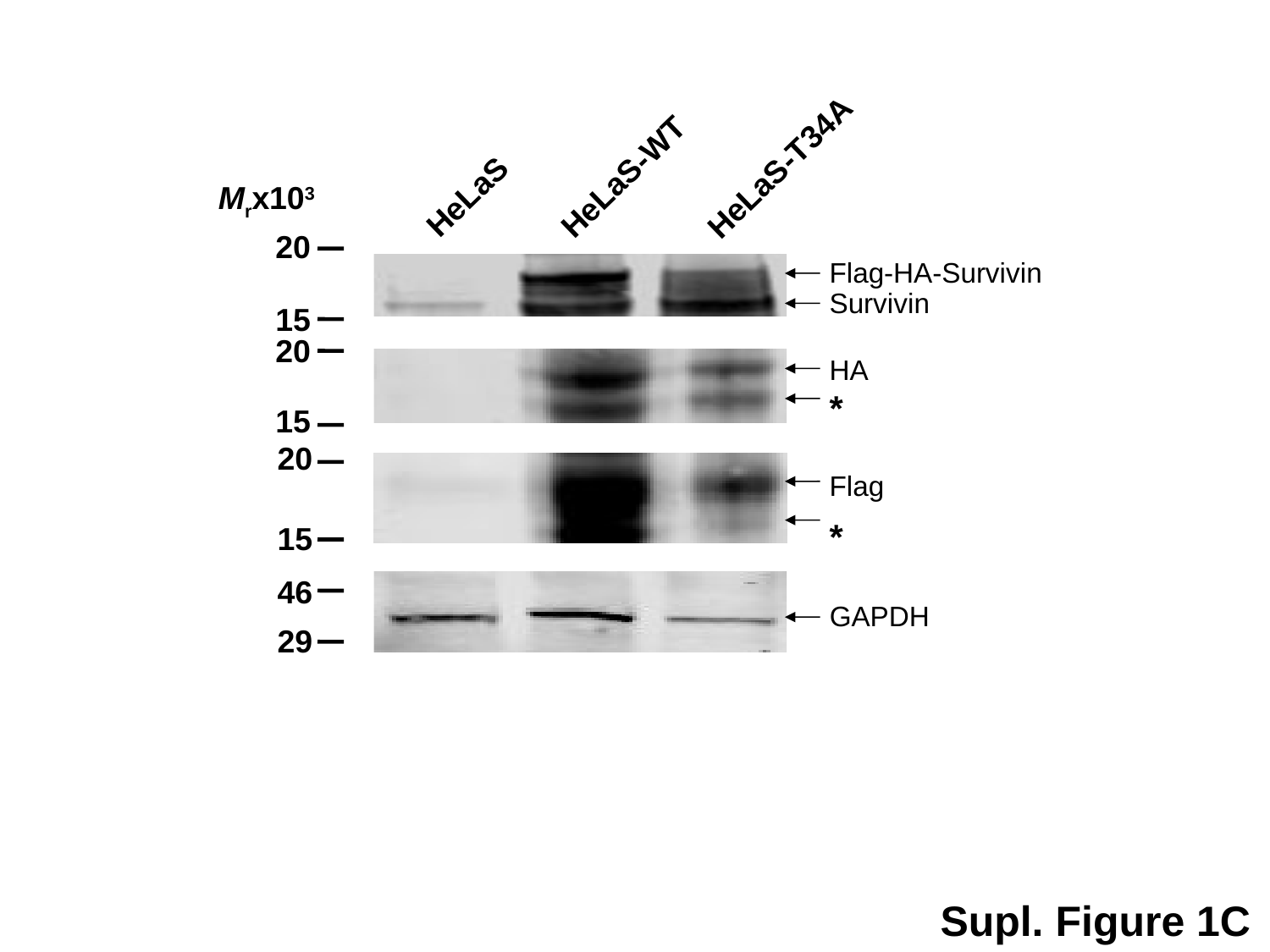

HeLaS-T34A
HeLaS-WT
HeLaS
Mrx103
20
15
Flag-HA-Survivin
Survivin
20
15
HA
*
20
15
Flag
*
46
29
GAPDH
Supl. Figure 1C

## Slide 4
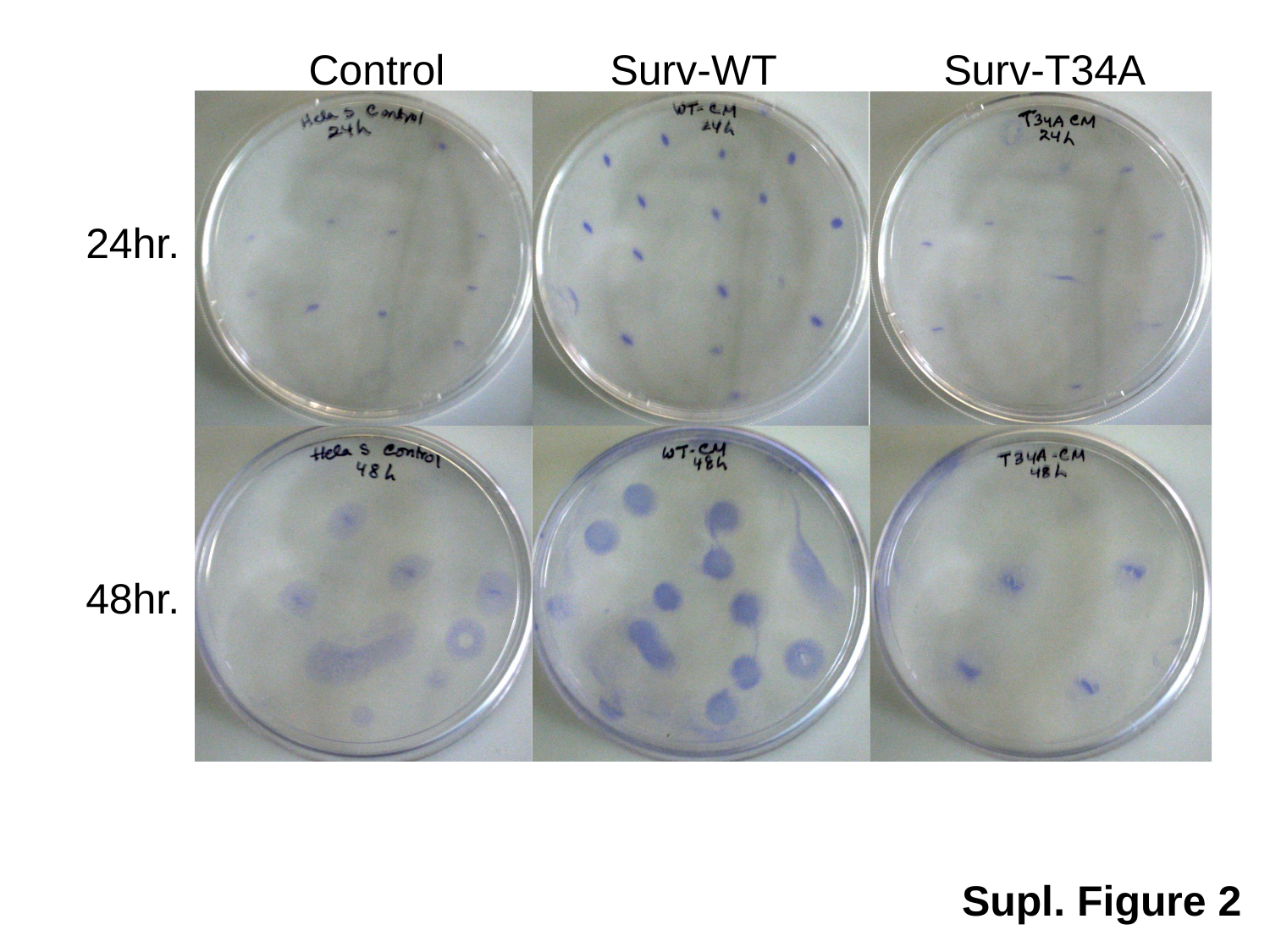

Control
Surv-WT
Surv-T34A
24hr.
48hr.
Supl. Figure 2

## Slide 5
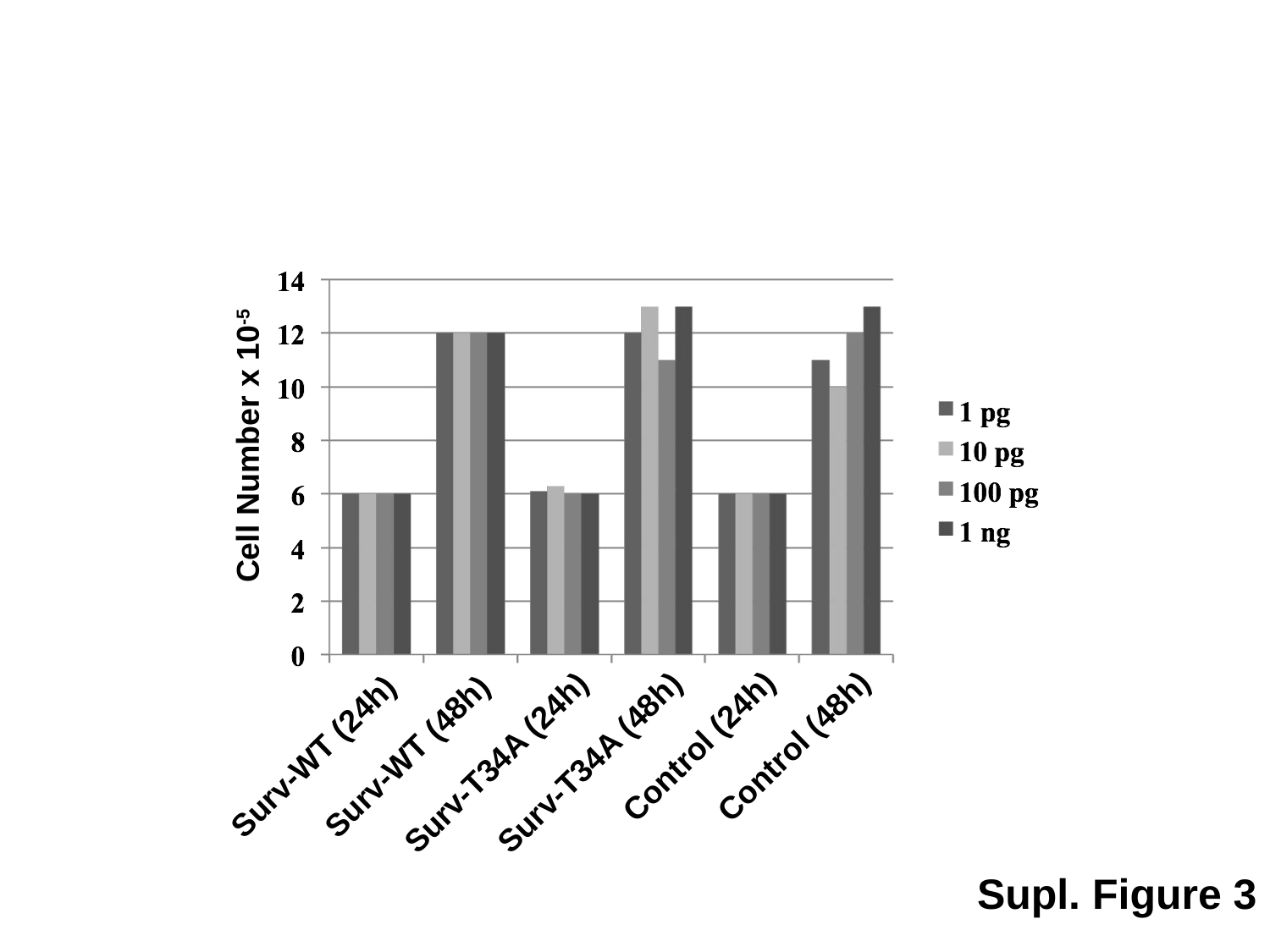

Cell Number x 10-5
Control (24h)
Control (48h)
Surv-WT (24h)
Surv-WT (48h)
Surv-T34A (24h)
Surv-T34A (48h)
Supl. Figure 3
