# Supplementary material for: Extracellular, cell-permeable survivin inhibits apoptosis while promoting proliferative and metastatic potential
Source: Br J Cancer. 2009 Mar 17;100(7):1073–86. doi: 10.1038/sj.bjc.6604978 (PMC2669990; doi:10.1038/sj.bjc.6604978)
Supplement: Supplementary Figure Legends [file 6604978x2.doc]

**Supplemental Figure 1.** HeLa cells express the Flag-HA-Survivin fusion protein. Wild type survivin **(A)** and T34A mutant survivin **(B)** transformed HeLa cells were selected four times using magnetic beads conjugated to the IL-2 Receptor antibody. Cells were fixed and stained using HA and Flag antibodies as described in the methods and materials. Magnification x1000. **(C)** HeLa cells that expressed the stable Flag-HA-Survivin fusion protein were harvested and analyzed by Western blotting with antibodies against survivin, HA, and Flag in order to support the immunohistochemical findings. As blots were often first probed with antibodies to survivin and then with antibodies to Flag or HA, a doublet band (indicated by arrows) occurs with survivin being 16.5 KDa and the Flag/HA retarding the band to ~18 KDa. A third band, labeled * often appears after probing with the antibodies to Flag and HA whose identity is yet unknown to us. GAPDH blotting was used as loading control. Molecular-weight markers in kilodaltons (KDa) are shown on the left.

**Supplemental Figure 2.** Surv-T34A conditioned medium induces tumor cell apoptosis while Surv-WT conditioned medium promotes cell growth. Cervical Carcinoma HeLa cells grown in the presence of either Surv-T34A or Surv-WT conditioned medium for 24 and 48 hours, were fixed with methanol and stained with crystal violet allowing visualization of the enhanced cellular proliferative effects of Surv-WT or the killing/growth repressive effects of Surv-T34A. Plates shown are representative of one of two independent experiments with comparable results.

**Supplemental Figure 3.** Recombinant survivin does not affect cell growth (Surv-WT) or life (Surv-T34A). HeLa cells were grown in increasing quantities of recombinant Surv-WT or Surv-T34A protein (1pg, 10pg, 100pg and 1ng) for 24 and 48 hours. Cells were harvested, stained with Trypan blue and counted.
